# Diagnosis of frailty and implications on surgical process in the elderly: A narrative review

**DOI:** 10.1097/EA9.0000000000000041

**Published:** 2023-11-23

**Authors:** Paola Aceto, Chiara Schipa, Ersilia Luca, Chiara Cambise, Claudia Galletta, Concezione Tommasino, Liliana Sollazzi

**Affiliations:** From the Dipartimento di Scienze dell’Emergenza, Anestesiologiche e della Rianimazione, Fondazione Policlinico Universitario A. Gemelli IRCCS, Rome, Italy (PA, CS, EL, CC, CG, LS), Dipartimento di Scienze biotecnologiche di base, cliniche intensivologiche e perioperatorie, Università Cattolica del Sacro Cuore, Rome, Italy (PA, LS) and Università degli Studi di Milano, Milan, Italy (CT)

## Abstract

Longevity has increased the proportion of the elderly in the population, and as a result ageing has become the leading factor for diseases such as cerebrovascular and cardiovascular disorders. It also makes surgical procedures more complex with potential life-threatening complications. In order to further investigate the role of ageing in modern healthcare, the term ‘frailty’ has been proposed to describe a condition of reduced functional reserve that leads to an increased risk of adverse health outcomes. The aim of this study was to review the pathophysiology of frailty and to highlight the most important tools to diagnose it, and their ability to predict the postoperative outcome. There are two major conceptual models that provide guidance for the detection of frailty: the Fried Phenotype Model and the Cumulative Deficit Model. These two main models have provided a base from which the assessment of frailty has developed. Two frailty assessment tools, the modified frailty index and the simplified frailty index play a key role in the preoperative setting because of their predictive power for postoperative risk quantification. Assessments of independence and/or cognitive function represent the main components that an ideal frailty tool should have to identify elderly people who are at risk of postoperative functional and cognitive deterioration. Cognitive impairment undoubtedly has a high association with frailty, but cognitive status is not included in many frailty assessments. In this regard, comprehensive geriatric assessment is a more complete evaluation tool, and it should be used whenever a frailty tool screening gives a positive result. Finally, frailty assessment is useful to explore the cumulative effect of comorbidities on the ageing patients’ functional reserves and to identify the appropriate level of in-hospital and postdischarge care.

**KEY POINTS**

- Frailty increases the peri-operative risk of morbidity and mortality in the elderly.
- Performing comprehensive geriatric assessment is crucial as even the best frailty predictors for postoperative complications lack careful evaluation of cognitive impairment and independence.
- Following positive screening for frailty, regardless of the screening tool used, elective surgical patients should be referred to a peri-operative frailty team expert for comprehensive geriatric assessment. The frail patient should undergo such specific preoperative treatments as are indicated by the resources available.

## Introduction

Due to the steady growth in the number of the elderly, old age has a key role in increasing the risk of adverse health outcomes in most clinical settings including surgery.^1^ Age is the primary risk factor for numerous morbidities such as cerebrovascular and heart disorders, and in association with surgical procedures may be life-threatening. Experts in gerontology agree there is a distinction between biological and chronological age.^2^ While chronological age relates to how long a person has existed, biological age does not increase at the same rate for everyone. The latter is more predictive than the former in determining an individual's ability to cope with any severe stress because it is the product of pathophysiological ageing processes, comorbidities and hereditary variables.^2^

The term ‘frailty’ has been introduced to resolve the discrepancy between chronological and biological age. Frailty is a multidimensional syndrome related to the accumulation of age-related and disease-related deficits. It refers to a condition of reduced functional reserve that leads to a vulnerable state with an increased risk of adverse health outcomes when exposed to both endogenous and exogenous stressors. Hence, frailty increases the peri-operative risk of morbidity and mortality in the elderly, now a daily challenge for anaesthesiologists.^3^

The aim of this study was to review the pathophysiology of frailty and to highlight the most important tools for its diagnosis and their ability to predict the postoperative outcome.

## Postoperative risk in frail elderly patients

Surgery is a challenge for the frail, whose conditions may become a threat to life, as every injury, even if harmless, might be lethal. Over the last few years, the association between frailty and postoperative outcomes in many surgical specialties has been a ‘hot” topic.^4^ Although the distribution of frail patients varies significantly across surgical specialties, mortality patterns are similar for low-intensity, moderate-intensity, and high-intensity specialties, making frailty an independent risk factor for mortality in all surgical specialties. Even low-intensity surgery such as minor plastic, orthopaedic, or otolaryngology procedures, are associated with significant mortality in the frail.^5^ The poor outcomes in frail surgical patients are strictly related to their baseline conditions; 95% of frail elderly patients reach hospital with one or more comorbidities.^6^ Among the many pathological conditions, cognitive impairment plays a key role in mortality, functional decline and postoperative delirium, all of which are associated with significant negative outcomes such as greater surgical complication rates, falls and discharge to long-term care facilities rather than home.^7^

## Physiological changes in the elderly: where does frailty originate?

Frail individuals are a distinct clinical group because their physiology is not a linear extension of age. They represent a challenge for healthcare. Frail patients experience a faster functional decline in numerous organs, leading to a global decline. Multimorbidity significantly contributes to frailty, which may in turn facilitate the development of chronic disease.^8,9^ It has been proposed that frailty can accelerate functional decline and regulate the effect of multimorbidity on health outcomes.^10,11^ Although the reasons of frailty are unknown, changes in body composition are striking, with fat mass taking the place of lean mass due to various endocrine dysfunctions and decreased reactivity to thyroxin, renin and aldosterone.^12^ While fat accumulation increases, muscle mass tends to decrease, reducing heat output and predisposing to hypothermia.^13^ Dehydration, malnutrition and altered drug metabolism result from changes in plasma protein composition along with a reduction in total body water. The impact of many drugs may change because of the combined decrease in hepatic clearance and increased volume of distribution.^14^ All pharmacokinetic and pharmacodynamic changes are particularly relevant for central nervous system (CNS) active drugs. Reduced cholinergic reserves in the elderly result in extreme sensitivity to anticholinergic drugs, and consequently we need to pay particular attention to multiple prescribing, given that the anticholinergic mechanism is common to a number of medications. In the elderly, polypharmacy contributes to cognitive impairment and physical frailty.^15^ Opioids are often combined with benzodiazepines and this combination can contribute to greater mortality.^16^ The elderly have a lower cardiac reserve because of physiological changes such as arterial stiffness and decreased sensitivity to beta-adrenergic transmission.^17^ A decrease in myocytes is associated with an increase in the number of fibroblasts, resulting in hypertrophy and interstitial fibrosis. This leads to lower myocardial compliance, which affects both diastolic relaxation and systolic contraction.^18^ As glandular epithelial cells, ciliary function, and mucus production are compromised, pulmonary clearances are also affected, putting elderly patients at a higher risk of respiratory infections.^19^ Both parenchyma and blood flow are compromised in the liver and this has an impact on drug metabolism. The loss of nephrons and the lowering of parenchymal blood flow in the kidney contribute to a decrease in glomerular filtration rate, which affects both medication metabolism and the ability to dilute or concentrate urine.^20,21^

All these changes represent the physiological ageing process and are not necessarily part of the disorder of frailty. Currently, there is no consensus on the pathophysiology of frailty, and over time various theories have been suggested to explain its development. Genomic instability, combined with DNA damage caused by cell cycle arrest, stem cell depletion and slowed intercellular communication, retard cell regeneration and metabolism.^22^ Malnutrition is a major risk factor in the development of frailty. Even during hospitalisation, elderly people frequently have low intake, resulting in vitamin deficiencies. Muscles will be catabolised to supply energy if there are insufficient proteins and lipids to maintain organ function and muscle activity, resulting in sarcopenia. Even the inflammatory response may be involved in the pathogenesis. The inflammatory indicators (C reactive protein, IL-6) rise, the immune system responds abnormally, and vitamin D and muscle strength fall.^23^ All the described hallmarks of ageing discussed above may contribute in different proportions to the functional decline of the frail, but none of them can be counted as a specific marker.

## Tools for frailty screening: the state of the art

**FIGURE 1:**
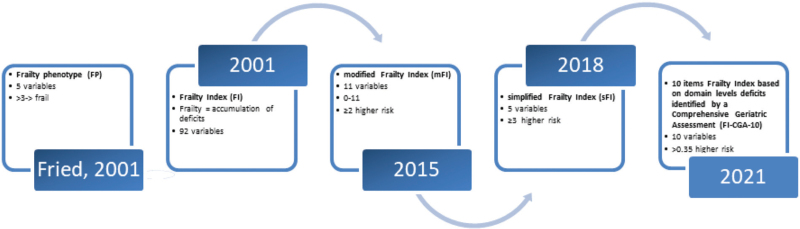
Evolution of frailty screening tools.

Many tools have been created over the years to evaluate frailty-related complications (Fig. 1). There are two major conceptual models that provide guidance for the detection of frailty: the Fried Phenotype Model and the Cumulative Deficit Model. These two main models are the source of several frailty assessment tools.^24,25^

Fried introduced the frailty phenotype in 2001, after gathering data from over 5000 patients; frailty manifests as a decline in five components including lean body mass, strength, endurance, walking performance and activity level. The presence of each sign and symptom was given a score from 0 to 5, and patients with at least three of these criteria were classified as frail.^24^ The Timed Up and Go Test (TUGT) and walk assessment test is a validated technique for monitoring mobility and risk of falling in the elderly as walking ability is crucial. The individual is first asked to stand up from a chair (which should not be leaning against a wall), walk 3 m, turn around, walk back to the chair and sit down. The TUGT performance is then rated on a scale of 1 to 5, with 1 indicating ‘normal function’ and 5 indicating ‘severely abnormal function’ based on the observer's perception of the individual's risk of falling.^26^ The walking test reveals a problem with gait. A sluggish (even if nonspecific) stride can indicate persistent cerebrovascular and neurological disorders. Due to the altered processing of information, a reduction in the performance of the prefrontal area, a region particularly prone to hypertensive insults, adds to a slow speed.^27^ Makary *et al.* demonstrated in 2010 that higher preoperative risk determined by Fried's criteria was related to an increased rate of problems and longer and more frequent hospitalisation. Due to its inaccuracy and lack of assessment of cognitive function, this sarcopenia-like model was challenged and gradually replaced.^28^

In 2001, Mitnitski *et al.*^29^ designed a different system to assess well being, defining frailty as an accumulation of deficits and laying the groundwork for the frailty index (FI). Starting from 400 variables, they selected binary variables with a scoring system based on 92 variables, including the symptoms associated with deficits (such as sleep and memory abnormalities, depression), signs (such as tremor), laboratory alterations (of urea, creatinine), comorbidities and disabilities.

More recently, Kim *et al.* designed a simplified version with 11 morbidity variables (including diabetes mellitus, hypertension and several cardiovascular diseases, functional capability and chronic obstructive pulmonary disease), which are part of the current modified Frailty Index (mFI or mFI-11).^30^ The final score ranges between 0 and 11, where 0 represents no frailty and 11 is the highest stage of frailty. This deficit accumulation model has become very popular for clinical research, particularly when data are extracted from medical records. In many multicentre prospective studies, elevated mFI was associated with increased infections, anastomotic leaks, complications and mortality.^31–34^ In many different studies, a higher mFI score was the strongest predictor for mortality and complications, after age and ASA classification were taken into account.^31–33^ Moreover, an association between mFI and unplanned re-admission to the hospital within 30 days has been recently demonstrated. More than 200 000 thousand patients undergoing orthopaedic (42%), abdominal (39%) and vascular (18%) surgery without age limits were studied. The most frequent comorbidities were treated hypertension, diabetes and ischaemic heart disease. The mFI increased with age and ASA classification, but it was still the strongest predictor of mortality, complications and re-admission.^34^ In 2018, a further simplification of the mFI was proposed by reducing the FI items from 11 to 5 (simplified FI, sFI), demonstrating similar predictivity for mortality and postoperative complications after any type of surgery.^35^

The usefulness of both mFI and sFI was also demonstrated in major urological surgery with mFI ≥ 2 or sFI ≥ 3 identifying patients at significantly higher risk of adverse short-term postoperative outcomes.^36^

Daily life functional independence represents a single item of mFI. This aspect could be more extensively explored by evaluating the so-called Activities Daily Living score (ADL).^37^ In 406 surgical geriatric patients (age >80 years), a high ADL score proved to be an independent risk factor for mortality 5 years after surgery. Patients with preserved independence in ADL showed survival rates similar to the general population, while more dependent patients had a higher mortality rate, with the loss of independence representing the first insult whereas the surgery was the second.^38^ Postoperative complications are more frequent in cases of preoperative functional dependence and play a key role in this combined effect, leading to a worse outcome and more frequent postoperative hospitalisation. The loss of independence often correlates with sarcopenia and muscle weakness.^39,40^ Despite not being included in mFI, sarcopenia represents one of the items of the Fried phenotype criteria and is associated with poorer postoperative outcomes. A recent meta-analysis identified sarcopenia as a negative predictor of postoperative outcomes in older patients undergoing gastrectomy.^41^ Frailty is not always linked to sarcopenia, but it leads to reduced body responsiveness to harmful events. Patients with chronic inflammation often experience catabolism and sarcopenia.^42^

Despite limited research on the treatment of sarcopenia, defined as loss of muscle mass, strength and function,^41^ strategies to ameliorate it could reduce health costs, improving short-term and long-term surgical outcomes in the elderly. Physical exercise is effective in nonsurgical cohorts. The first step in reducing muscle loss is to identify the high-risk patients and treat them before the surgical insult, within the available time.^43^

There are many other tools, with variable feasibility for anaesthesiologists. Among these, the Risk Analysis Index (RAI) that explores 14 items and a range from 0 to 81, in less than a minute, deserves a special mention. An elevated RAI up to 25 correlated well with mortality, prolonged hospital stay and increased rehabilitation needs after surgery.^44^

The Geriatric 8 (G8) is another quick-to-apply and easy-to-read screening tool, a feasible 8-question survey that allows clinicians to assess the degree of frailty in less than 1 min. A correlation between a score lower than 12 and postoperative mortality has been recently demonstrated.^45^ A recent meta-analysis demonstrated that FRAIL scale, based on fatigue, resistance, ambulation, illness and loss of weight, was able to predict 30-day mortality, 6-month mortality, postoperative complications and postoperative delirium.^46^

The Time to Up and Go (TUG Test) also showed a significant correlation with high-grade postoperative complications but was not always applicable in clinical practice.^47^

Frailty surrogate measures, such as hand strength, are also widely used in peri-operative medicine. Grip strength is a simple and inexpensive risk screening tool associated with all-cause mortality, adverse peri-operative events and discharge to another care facility rather than home, in patients with vascular disease.^48^ Even if some of the tools that measure frailty, such as Timed Up & Go, grip strength, are not validated, they are currently used in clinical practice, as suggested,^3^ as useful tools to screen patients requiring further evaluations like comprehensive geriatric assessment (CGA).

### Clinical practice: easy and quick tools to identify frailty

Currently, the international guidelines for frail patients recommend a multidisciplinary approach and the use of a frailty score, such as the Clinical Frailty Scale (CFS), the electronic frailty index (eFI) or the Edmonton Frailty Scale (EFS). Preoperative screening could be carried out by surgical or other clinicians first visiting the patients. Frail elective surgical patients (reporting CFS ≥5) should be referred to a peri-operative frailty team expert. In the absence of a frailty team, the clinician should identify and make the required interventions, for example, prehabilitation.^49^

According to Buta *et al.*,^50^ published reports indicate that the most used frailty tool is the Physical Frailty Phenotype scoring system, based on the ‘first-minute impression’, that clinicians use, more or less consciously, when evaluating patients in a face-to-face setting during the preoperative visit. This has been then incorporated into the CFS, which identifies nine categories of patients from very fit to terminal. The CFS scores range from 1 (very fit) to 9 (terminally ill) based on pictorial representations and accompanying descriptors of functional status: nonfrail (CFS 1 to 4), mild to moderately frail (CFS 5 to 6) and severely frail (CFS 7 or above). This scale has been used to predict death or the need for long-term care and has a strong correlation with mFI.^51^ In a more recent meta-analysis comparing the different frailty scoring systems, CFS came out as the strongest predictor of discharge to further care and mortality; unfortunately, as an important limitation, CFS is validated only after CGA.^52^

CGA is a very comprehensive evaluation tool that in its original version requires a multidimensional and multidisciplinary assessment process requiring the measurement of frailty through the assessment of concomitant disorders, cognitive, psychological, nutritional and functional status, taking into account polypharmacy, social support and geriatric syndrome. Feasibility represents the main limitation of CGA as it takes at least 30 min to complete and is not always easy to employ in different clinical situations limiting its use as a preoperative assessment tool.^53,54^

The guidelines from the Association of Anaesthetists of Great Britain and Ireland recommend evaluating some components of the CGA in addition to the classic preoperative ASA classification, with particular emphasis on the evaluation of cognitive status and mobility. This improves the prediction of postoperative complications compared with the ASA classification alone.^55,56^

The Edmonton Frail Scale (EFS), a small version of the frailty index, includes other relevant domains such as social support, mood, polypharmacy, and cognition, and seems to be feasible for daily use by nongeriatricians for predicting postoperative delirium (POD).^57,58^

Frailty may represent a risk factor for POD, and it should always be screened before surgery, as POD is a frequent complication in the elderly and prolongs the time to discharge home and mortality.^59^ Ensuring adequate frailty screening may lead clinicians to implement strategies to enhance POD prevention and possibly reduce related adverse outcomes.^42,59^ The decision-making process on whether to intervene or not must include a screening test to identify frailty before elective surgery. Preoperative diagnosis of frailty is useful for predicting postoperative outcome and it allows planning of specific measures to improve outcomes in frail geriatric patients, such as the appropriate levels of in-hospital (ward or ICU) and postdischarge care (home or nursing home).^36^

The number of publications on measuring frailty has increased in recent years, but there is still no consensus on how frailty should be measured and no standard comprehensive tool for screening frailty. Measurement tools need sensitivity and specificity when detecting frailty, but they should also be feasible and quick if they are to be used in routine clinical practice. The faster and easier it is to use these screening tools, the greater their ability to identify frail patients, predict peri-operative complications and possibly improve the outcome. We used three recent meta-analyses published in 2020 and 2022, to build a table with the main characteristics of every tool (see Table 1).^51,60,61^ Aucoin *et al.*^52^ identified 45 articles using 35 different tools for measuring frailty that were good predictors for postoperative outcome in terms of mortality and hospitalisation. In particular, they found that CFS is the best tool for identifying mortality and discharge to further care, with the highest reported measures of feasibility, while EFS is excellent for the prediction of postoperative complications, and FP for POD.^52^ The EFS was developed as a practical tool to be used by healthcare providers without specialised geriatrics training. It is user-friendly and requires less than 5 min to administer. The EFS has been shown to be a reliable measure of frailty as the results were in good agreement with geriatricians’ clinical impressions based on a complete history and physical examination.^62^ In elderly people, EFS provides similar results compared with other screening tools.^63^ When used as a screening tool in the Caucasian population, EFS has also been found to predict peri-operative risk.^57^ Chan *et al.*^61^ explored in 13 articles the importance of preoperative frailty assessment, regardless of a specific frailty measurement tool, to predict mortality, length of stay and discharge to healthcare facility. Shaw *et al.*^60^ identified 71 studies of frailty tools and described how adverse postoperative complications and mortality after elective cancer surgery correlate with frailty, suggesting the usefulness of frailty tools in the preoperative setting.

**Table 1:** Main characteristics of the most popular frailty assessment tools

Tools	Time-to-complete	Variables	Range score	Cut-off	Year	Predictivity	Suitability	Feasibility
FP	5 to 20 min (average of 10 min)	5 domains	0 to 5	3	2001 (Fried 2001)	Mortality in geriatric cancer surgical and orthopaedic patients (Gleason 2017, Kapoor 2017, Pelavski 2017), in cardiac surgery (Huded 2016, Lytwyn 2017, Afilalo 2010). Postoperative complications (Goldstein 2020, Makary 2010, Courtney-Brooks 2012, Revenig 2015, Revenig 2013, Andreou 2018, Brown 2016, Cooper 2016, Kapoor 2017, Katlic 2019, Jha 2017) POD (Gleason 2017, Khan 2016, Leung 2011, Kua 2016) LOS (Ad 2016, Brown 2016, Andreou 2018, Jha 2017, Khan 2016, Kim 2016, McIsaac 2018, Lytwyn 2017, Wang 2018, Sikder 2018, Huded 2016, Gleason 2017, Cooper 2016, Pelavski 2017)	3	2
CFS 2.0	<1 min (44 s)	9 points	1 to 9	4	2007 (Rockwood, 2005)	Mortality and complications in older general and cardiac surgery (Afilalo 2017, Goeteyn 2017, McGuckin 2018, Donald 2018, Rodrigues 2017, Reichart 2018, Tipping 2020, Misawa 2020) LOS (Donald 2018, McIsaac 2018, Goeteyn 2017, McGuckin 2018, Wang 2018, Rodrigues 2017, Li 2018, Yamada 2021)	2	3
FI	10 min	Max 40 health deficits	0 to 1	0.25	2001 (Mitnitski, 2001)	Post operative complications (Saxton 2011, Cooper 2016, Joseph 2016, Orouji-Jokar 2016, Lin 2017, Lu 2018) LOS (Joseph 2016, Li 2018)	1	1
EFS	5 min	11 items	0 to 17	8	2000	Mortality and postoperative complications (Kiss 2020, Dasgupta 2009, Kua 2016, Kovacs 2017, Amabili 2019) POD (Partridge 2015, Kua 2016)	2	1
CGA	Minimum 30 min	9 criteria	Process of care	Reaching the cut-off value in at least two CGA parameters	1993 (Stuck, 1993)	Mortality, disability, and institutionalisation (Parker 2018, Miller 2022) Mobility (Taraldsen 2014) LOS (Vidan 2005, Partridge 2017)	2	1
mFI-11	5 min	11 items	0 to 11	2	2015 (Kim, 2015)	Mortality (Hamidi 2019) Postoperative complications (Wahl 2017, Lakomkin 2019, Pitts 2019, Voskam 2020, Giannini 2019, Osaki 2020) and trauma complications (Tracy 2020)	2	3
sFI-5	3 min	5 items	0 to 5	3	2018 (Subramaniam 2018)	Mortality in trauma (Tracy 2020) and postoperative complications (Gearhart 2020, Miller 2020, Subramaniam 2021)	2	3
FI-CGA-10	15 min	10 items	0 to 1	0.35	2021 (Nishijima 2021)	Adverse outcomes in older adults with various cancer types.	0	2
RAI	1 min	14 items	0 to 81	25	2017	Mortality, extended stay, and increased rehabilitation needs after surgery (Isharwal 2017, Dittman 2022, Al-Damluji 2022, Abdelfatah 2023, Grudzinski 2022) Postoperative outcomes in a dose-dependent manner (Tse 2021) Long-term death (Rothenberg 2020)	1	3
G-8	1 min	8 questions	0 to 17	14	2012	Major and minor post-operative complications (Anic 2022, Nakayama 2022, Yajima 2022, Zennami 2022,)	1	3
TUG	90 s	1	12 to 85	12	1991	Mobility, morbidity and complications (Sasaki 2010, Huisman 2014, Schmidt 2018, Hsieh 2020, Komodikis 2020, Rabelo 2023)	1	2
FRAIL-scale	<10 min	5 items	0 to 5	3	2008	Complications, Mortality (Gleason 2017, Miguelena-Hycka 2019, Valdatta 2019, Arteaga 2020, Yin 2021, Berastegui 2020, Pedemonte 2021, Torres-Perez 2021) LOS (Mahanna-Gabrielli 2020, Duchnowski 2020) POD (Acedo 2020, Susano 2020)	2	3

A description of the time required for completion, number of variables taken into consideration, range and cut-off for the definition of fragility, year of introduction, and prediction value in term of mortality, peri-operative complications, POD, length of stay, other outcomes, as available in three recent meta-analyses has been detailed for each tool. Please see Supplementary materials for references cited in this table. Suitability and feasibility from 0 to 3: 0 insufficient, 1 (sufficient), 2 (good), 3 (excellent). CFS, Clinical Frailty Scale; CGA, Comprehensive Geriatric Assessment; ED, Emergency Department; EFS, Edmonton Frailty Scale; FI, Frailty Index; FI-CGA-10, 10-item Frailty Index based on domain-level deficits in a standardised CGA; FP, Frailty Phenotype; G-8, Geriatric-8; LOS Length of Stay; mFI-11, modified Frailty Index with 11 variables; POD, postoperative Delirium; RAI, Risk Analysis Index; sFI-5, simplified Frailty Index with 5 items; TUG, Timed Up and Go.

In this context, the FI-CGA-10, a frailty index based on domain-level deficits identified through a comprehensive geriatric assessment that represents a simplification of FI-CGA, has been developed but not yet used in elderly surgical patients.^64^

Some authors have identified frailty as an independent predictor for adverse outcomes for patients with SARS-CoV2 infection. In a recent study, including 785 older COVID-19 patients, CFS-defined frailty was independently associated with mortality regardless of severity while mild-to-moderate frailty was related to re-admission.^65^ Ferrara *et al.* revealed that one out of every three older patients previously hospitalised with COVID-19 had a worse CFS score over a 6-month median follow-up period. Therefore, not only frailty evaluation may assist clinicians in better stratifying the risk of mortality for older COVID-19 patients but SARS-Cov2 infection should also be counted as an aggravating comorbidity in elderly patients with preexisting frailty.^66^

This review is an evidence-based resource of practical frailty screening tools that are available to the anaesthesiologist who wishes to predict postoperative outcome in the elderly. The most used frailty screening tools were scored by authors for suitability and feasibility in the preoperative setting based on recent findings (Table 1). Preoperative optimisation plays a key role in elective surgery, as demonstrated in the ERAS protocol, in increasing cardio-pulmonary function, peripheral perfusion and adaptive stress response.^67^ For elective surgery, identifying frail patients is essential if individualised prehabilitation programs are to be implemented.^3^

## Conclusion

Quantifying the risk of frailty can potentially improve postoperative outcomes but only when frailty is integrated into the medical decision-making process.

mFI or sFI may represent the best options because of their valuable suitability and feasibility. Assessments of independence and/or cognitive function represent the main components of an ideal frailty tool that seeks to identify elderly people who are at risk of postoperative functional and cognitive deterioration. The CGA is a more comprehensive evaluation tool but is perhaps too lengthy for routine clinical practice, and should possibly be used when a frailty screening tool gives a positive result. This approach needs to be further investigated in future studies.

## Supplementary Material

Supplemental Digital Content
