# Supplementary material for: Diagnosis of frailty and implications on surgical process in the elderly: A narrative review
Source: Eur J Anaesthesiol Intensive Care. 2023 Nov 23;2(6):e0041. doi: 10.1097/EA9.0000000000000041 (PMC11798398; doi:10.1097/EA9.0000000000000041)
Supplement: Supplemental Digital Content [file ejaic-2-e0041-s001.docx]

References list for Table 1 (in alphabetic order)

- Abdelfatah E, Ramos-Santillan V, Cherkassky L, Cianchetti K, Mann G. High Risk, High Reward: Frailty in Colorectal Cancer Surgery is Associated with Worse Postoperative Outcomes but Equivalent Long-Term Oncologic Outcomes. *Ann Surg Oncol*. 2023.
- Acedo PS, Córdoba IE, Ripa CZ, Cabezón JH, Castilla AT. Prospective study of factors associated with postoperative delirium after urgent abdominal surgery. *Cir Esp* (Engl Ed). 2020;**98**:450–455.
- Ad N, Holmes SD, Halpin L, et al. The Effects of Frailty in Patients Undergoing Elective Cardiac Surgery. *J Card Surg* 2016; **31**:187-194.
- Afilalo J, Eisenberg MJ, Morin J-F, et al. Gait speed as an incremental predictor of mortality and major morbidity in elderly patients undergoing cardiac surgery. *J Am Coll Cardiol* 2010; **56**:1668-1676.
- Afilalo J, Lauck S, Kim DH, et al. Frailty in Older Adults Undergoing Aortic Valve Replacement: The FRAILTY-AVR Study. *J Am Coll Cardiol* 2017; **70**:689-700.
- Al-Damluji MS, Smolderen KG, Meng C, Dai F, Nanna MG, Sumpio B, Henke P, Mena-Hurtado C. Frailty and outcomes following revascularization of lower-extremity peripheral artery disease: Insights from the Vascular Quality Initiative (VQI). *Vasc Med*. 2022;**27**(3):251-257.
- Amabili P, Wozolek A, Noirot I, et al. The Edmonton Frail Scale Improves the Prediction of 30-Day Mortality in Elderly Patients Undergoing Cardiac Surgery: A Prospective Observational Study. *J Cardiothorac Vasc Anesth* 2019; **33**:945-952.
- Andreou A, Lasithiotakis K, Venianaki M, et al. A Comparison of Two Preoperative Frailty Models in Predicting Postoperative Outcomes in Geriatric General Surgical Patients. *World J Surg* 2018; **42**:3897-3902.
- Anic K, Varchola J, Schmidt MW, Schwab R, Linz VC, Schmidt M, Hardt R, Hartmann EK, Ruckes C, Hasenburg A, Battista MJ. Influence of interdisciplinary frailty screening on perioperative complication rates in elderly ovarian cancer patients: results of a retrospective observational study. *Arch Gynecol Obstet*. 2022.
- Arteaga AS, Aguilar LT, González JT, et al. Impact of frailty in surgical emergencies. A comparison of four frailty scales. *Eur J Trauma Emerg Surg*. 2020;**47**:1613–1619.
- Berastegui Garcia E, Camara Rosell ML, Moret Ruiz E, et al. The impact of frailty in aortic valve surgery. *BMC Geriatr*. 2020;**20**:426.
- Brown CH 4th, Max L, LaFlam A, et al. The Association Between Preoperative Frailty and Postoperative Delirium After Cardiac Surgery. *Anesth Analg* 2016; **123**:430-435.
- Chan R, Ueno R, Afroz A, et al. Association between frailty and clinical outcomes in surgical patients admitted to intensive care units: a systematic review and meta-analysis. *Br J Anaesth* 2022; **128**:258-271.
- Cooper Z, Rogers SOJ, Ngo L, et al. Comparison of Frailty Measures as Predictors of Outcomes After Orthopedic Surgery. *J Am Geriatr Soc* 2016; **64**:2464-2471.
- Courtney-Brooks M, Tellawi AR, Scalici J, et al. Frailty: an outcome predictor for elderly gynecologic oncology patients. *Gynecol Oncol* 2012; **126**:20-24.
- Dasgupta M, Rolfson DB, Stolee P, et al. Frailty is associated with postoperative complications in older adults with medical problems. *Arch Gerontol Geriatr* 2009; **48**:78-83.
- Dittman JM, Lavingia KS, Larson RA. Elevated Risk Analysis Index (RAI) Frailty Scores Are Independently Associated with Adverse Outcomes in Lower Extremity Surgical Revascularizations Similarly Across Genders. *Ann Vasc Surg*. 2022; **87**:47-56.
- Donald GW, Ghaffarian AA, Isaac F, et al. Preoperative frailty assessment predicts loss of independence after vascular surgery. *J Vasc Surg* 2018; **68**:1382-1389.
- Duchnowski P, Szymański P, Kuśmierczyk M, Hryniewiecki T. Usefulness of FRAIL scale in heart valve diseases. *Clin Interv Aging*. 2020;**15**:1071–1075.
- Fried LP, Tangen CM, Walston J, et al. Frailty in older adults: evidence for a phenotype. *J Gerontol A Biol Sci Med Sci* 2001; 56:M146-56.
- Gearhart SL, Do EM, Owodunni O, et al. Loss of Independence in Older Patients after Operation for Colorectal Cancer. *J Am Coll Surg* 2020; **230**:573-582.
- Giannini A, Di Donato V, Schiavi MC, et al. Predictors of postoperative overall and severe complications after surgical treatment for endometrial cancer: The role of the fragility index. *Int J Gynaecol Obstet* 2020; **148**:174-180.
- Gleason LJ, Benton EA, Alvarez-Nebreda ML, et al. FRAIL Questionnaire Screening Tool and Short-Term Outcomes in Geriatric Fracture Patients. *J Am Med Dir Assoc* 2017; **18**:1082-1086.
- Goeteyn J, Evans LA, De Cleyn S, et al. Frailty as a predictor of mortality in the elderly emergency general surgery patient. *Acta Chir Belg* 2017; **117**:370-375.
- Goldstein DP, Sklar MC, de Almeida JR, et al. Frailty as a predictor of outcomes in patients undergoing head and neck cancer surgery. *Laryngoscope* 2020; **130**:E340-E345.
- Grudzinski AL, Aucoin S, Talarico R, Moloo H, Lalu MM, McIsaac DI. Measuring the Predictive Accuracy of Preoperative Clinical Frailty Instruments Applied to Electronic Health Data in Older Patients Having Emergency General Surgery: A Retrospective Cohort Study. *Ann Surg*. 2022.
- Hamidi M, Zeeshan M, Leon-Risemberg V, et al. Frailty as a prognostic factor for the critically ill older adult trauma patients. *Am J Surg* 2019; **218**:484-489.
- Huded CP, Huded JM, Friedman JL, et al. Frailty Status and Outcomes After Transcatheter Aortic Valve Implantation. *Am J Cardiol* 2016; **117**:1966-1971.
- Huisman MG, van Leeuwen BL, Ugolini G, Montroni I, Spiliotis J, Stabilini C, de'Liguori Carino N, Farinella E, de Bock GH, Audisio RA. "Timed Up & Go": a screening tool for predicting 30-day morbidity in onco-geriatric surgical patients? A multicenter cohort study. *PLoS One*. 2014;**9**(1):e86863.
- Hsieh CY, Huang HY, Liu KC, Chen KH, Hsu SJ, Chan CT. Subtask Segmentation of Timed Up and Go Test for Mobility Assessment of Perioperative Total Knee Arthroplasty. *Sensors* (Basel). 2020;**20**(21):6302.
- Isharwal S, Johanning JM, Dwyer JG, Schimid KK, LaGrange CA. Preoperative frailty predicts postoperative complications and mortality in urology patients. *World J Urol*. 2017;**35**(1):21-26.
- Jha SR, Hannu MK, Newton PJ, et al. Reversibility of Frailty After Bridge-to-Transplant Ventricular Assist Device Implantation or Heart Transplantation. *Transplant Direct* 2017; **3**:e167.
- Joseph B, Zangbar B, Pandit V, et al. Emergency General Surgery in the Elderly: Too Old or Too Frail? *J Am Coll Surg* 2016; **222**:805-813.
- Komodikis G, Gannamani V, Neppala S, et al. Usefulness of Timed Up and Go (TUG) Test for Prediction of Adverse Outcomes in Patients Undergoing Thoracolumbar Spine Surgery. *Neurosurgery*. 2020; **86**(3):E273-E280.
- Kapoor A, Matheos T, Walz M, et al. Self-Reported Function More Informative than Frailty Phenotype in Predicting Adverse Postoperative Course in Older Adults. *J Am Geriatr Soc* 2017; **65**:2522-2528.
- Katlic MR, Coleman J, Khan K, et al. Sinai Abbreviated Geriatric Evaluation: Development and Validation of a Practical Test. *Ann Surg* 2019; **269**:177-183.
- Khan SA, Chua HW, Hirubalan P, et al. Association between frailty, cerebral oxygenation and adverse post-operative outcomes in elderly patients undergoing non-cardiac surgery: An observational pilot study. *Indian J Anaesth* 2016; **60**:102-107.
- Kim S, Brooks AK, Groban L. Preoperative assessment of the older surgical patient: honing in on geriatric syndromes. *Clin Interv Aging* 2015; **10**:13-27.
- Kim S, Marsh AP, Rustowicz L, et al. Self-reported Mobility in Older Patients Predicts Early Postoperative Outcomes after Elective Noncardiac Surgery. *Anesthesiology* 2016; **124**:815-825.
- Kiss R, Farkas N, Jancso G, et al. Determination of frail state and association of frailty with inflammatory markers among cardiac surgery patients in a Central European patient population. *Clin Hemorheol Microcirc* 2020; **76**:341-350.
- Kovacs J, Moraru L, Antal K, et al. Are frailty scales better than anesthesia or surgical scales to determine risk in cardiac surgery? *Korean J Anesthesiol* 2017; **70**:157-162.
- Kua J, Ramason R, Rajamoney G, et al. Which frailty measure is a good predictor of early post-operative complications in elderly hip fracture patients? *Arch Orthop Trauma Surg* 2016; **136**:639-647.
- Lakomkin N, Zuckerman SL, Stannard B, et al. Preoperative Risk Stratification in Spine Tumor Surgery: A Comparison of the Modified Charlson Index, Frailty Index, and ASA Score. *Spine* 2019; **44**:E782-E787.
- Leung JM, Tsai TL, Sands LP. Brief report: preoperative frailty in older surgical patients is associated with early postoperative delirium. *Anesth Analg* 2011; **112**:1199-1201.
- Li S, Nie Y, Zhan J, et al. The analysis of correlation between frailty index and postoperative complications of aged patients with nodular goiter. *Aging Med* (Milton) 2018; **1**:18-22.
- Lin H, Peel NM, Scott IA, et al. Perioperative assessment of older surgical patients using a frailty index-feasibility and association with adverse post-operative outcomes. *Anaesth Intensive Care* 2017; **45**:676-682.
- Lu J, Zheng H-L, Li P, et al. High preoperative modified frailty index has a negative impact on short- and long-term outcomes of octogenarians with gastric cancer after laparoscopic gastrectomy. *Surg Endosc* 2018; **32**:2193-2200.
- Lytwyn J, Stammers AN, Kehler DS, et al. The impact of frailty on functional survival in patients 1 year after cardiac surgery.  *J Thorac Cardiovasc Surg* 2017; **154**:1990-1999.
- Mahanna-Gabrielli E, Zhang K, Sieber FE, et al. Frailty is associated with postoperative delirium but not with postoperative cognitive decline in older noncardiac surgery patients. *Anesth Analg*. 2020;**130**:1516–1523.
- Makary MA, Segev DL, Pronovost PJ, et al. Frailty as a predictor of surgical outcomes in older patients. *J Am Coll Surg* 2010; **210**:901-908.
- McGuckin DG, Mufti S, Turner DJ, et al. The association of peri-operative scores, including frailty, with outcomes after unscheduled surgery. *Anaesthesia* 2018; **73**:819-824.
- McIsaac DI, Huang A, Wong CA, et al. Effect of Preoperative Geriatric Evaluation on Outcomes After Elective Surgery: A Population-Based Study. *J Am Geriatr Soc* 2017; **65**:2665-2672.
- McIsaac DI, Macdonald DB, Aucoin SD. Frailty for Perioperative Clinicians: A Narrative Review. *Anesth Analg* 2020; **130**:1450-1460.
- Miguelena-Hycka J, Lopez-Menendez J, Prada PC, et al. Influence of preoperative frailty on health-related quality of life after cardiac surgery. *Ann Thorac Surg*. 2019;**108**:23–29.
- Miller SM, Wolf J, Katlic M, et al. Frailty is a better predictor than age for outcomes in geriatric patients with rectal cancer undergoing proctectomy. *Surgery* 2020; **168**:504-508.
- Miller RL, Barnes JD, Mouton R, Braude P, Hinchliffe R. Comprehensive geriatric assessment (CGA) in perioperative care: a systematic review of a complex intervention. *BMJ Open*. 2022; **21**:12(10):e062729.
- Misawa N, Higurashi T, Tachikawa J, et al. Clinical impact of evaluation of frailty in endoscopic submucosal dissection for early gastric cancer in elderly patients. *Geriatr Gerontol Int* 2020; **20**:461-466.
- Mitnitski AB, Mogilner AJ, Rockwood K. Accumulation of deficits as a proxy measure of aging. *ScientificWorldJournal* 2001; **1**:323-36.
- Nakayama Y, Ohkoshi A, Ishii R, Higashi K, Nakanome A, Ogawa T, Katori Y. The geriatric-8 screening tool for predicting complications in older adults after surgery for locally advanced head and neck cancer with free flap reconstruction. *Eur Arch Otorhinolaryngol*. 2022; **279**:2565-2571.
- Nishijima TF, Shimokawa M, Esaki T, et al. A 10-Item Frailty Index Based on a Comprehensive Geriatric Assessment (FI-CGA-10) in Older Adults with Cancer: Development and Construct Validation. *Oncologist* 2021; **26**:e1751-e1760.
- Orouji Jokar T, Ibraheem K, Rhee P, et al. Emergency general surgery specific frailty index: A validation study. *J Trauma Acute Care Surg* 2016; **81**:254-260.
- Osaki T, Saito H, Shimizu S, et al. Modified Frailty Index is Useful in Predicting Non-home Discharge in Elderly Patients with Gastric Cancer Who Undergo Gastrectomy. *World J Surg* 2020; **44**:3837-3844.
- Parker SG, McCue P, Phelps K, et al. What is Comprehensive Geriatric Assessment (CGA)? An umbrella review. *Age Aging* 2018; **47**:149-155.
- Partridge JSL, Fuller M, Harari D, et al. Frailty and poor functional status are common in arterial vascular surgical patients and affect postoperative outcomes. *Int J Surg* 2015; **18**:57-63.
- Partridge JSL, Harari D, Martin FC, et al. Randomized clinical trial of comprehensive geriatric assessment and optimization in vascular surgery. *Br J Surg* 2017;**104**:679–87
- Pedemonte JC, Sun H, Franco-Garcia E, et al. Postoperative delirium mediates 180-day mortality in orthopaedic trauma patients. *Br J Anaesth.* 2021;**127**:102–109.
- Pelavski AD, De Miguel M, Alcaraz Garcia-Tejedor G, et al. Mortality, Geriatric, and Nongeriatric Surgical Risk Factors Among the Eldest Old: A Prospective Observational Study. *Anesth Analg* 2017; **125**:1329-1336.
- Pitts KD, Arteaga AA, Stevens BP, et al. Frailty as a Predictor of Postoperative Outcomes among Patients with Head and Neck Cancer. *Otolaryngol Head Neck Surg* 2019; 160:664-671.
- Rabelo LG, Bjornsdottir A, Jonsdottir AB, Einarsson SG, Karason S, Sigurdsson MI. Frailty assessment tools and associated postoperative outcomes in older patients undergoing elective surgery: A prospective pilot study. *Acta Anaesthesiol Scand.* 2023;**67**(2):150-158.
- Reichart D, Rosato S, Nammas W, et al. Clinical frailty scale and outcome after coronary artery bypass grafting. *Eur J Cardiothorac Surg* 2018; **54**:1102-1109.
- Revenig LM, Canter DJ, Kim S, et al. Report of a Simplified Frailty Score Predictive of Short-Term Postoperative Morbidity and Mortality. *J Am Coll Surg* 2015; **220**:904-11.e1.
- Revenig LM, Canter DJ, Taylor MD, et al. Too frail for surgery? Initial results of a large multidisciplinary prospective study examining preoperative variables predictive of poor surgical outcomes. *J Am Coll Surg* 2013; **217**:665-670.e1.
- Rockwood K, Song X, MacKnight C, et al. A global clinical measure of fitness and frailty in elderly people. *CMAJ* 2005; **173**:489-95.
- Rockwood K, Theou O. Using the Clinical Frailty Scale in Allocating Scarce Health Care Resources. *Can Geriatr J* 2020; 23:210-215.
- Rodrigues MK, Marques A, Lobo DML, et al. Pre-Frailty Increases the Risk of Adverse Events in Older Patients Undergoing Cardiovascular Surgery. *Arq Bras Cardiol* 2017; **109**:299-306.
- Rothenberg KA, George EL, Barreto N, Chen R, Samson K, Johanning JM, Trickey AW, Arya S. Frailty as measured by the Risk Analysis Index is associated with long-term death after carotid endarterectomy. *J Vasc Surg.* 2020;**72**(5):1735-1742.e3.
- Sasaki K, Senda M, Nishida K, Ota H. Preoperative time required for the timed "up and go" test in women with hip osteoarthritis could predict a deep venous thrombosis complication after total hip arthroplasty. *Acta Med Okayama*. 2010;**64**(3):197-201.
- Saxton A, Velanovich V. Preoperative frailty and quality of life as predictors of postoperative complications. *Ann Surg* 2011; **253**:1223-1229.
- Schmidt M, Eckardt R, Altmeppen S, Wernecke KD, Spies C. Functional impairment prior to major non-cardiac surgery is associated with mortality within one year in elderly patients with gastrointestinal, gynaecological and urogenital cancer: A prospective observational cohort study. *J Geriatr Oncol.* 2018;**9**(1):53-59.
- Shaw JF, Budiansky D, Sharif F, et al. The Association of Frailty with Outcomes after Cancer Surgery: A Systematic Review and Metanalysis. *Ann Surg Oncol* 2022; **29**:4690-4704.
- Sikder T, Sourial N, Maimon G, et al. Postoperative Recovery in Frail, Pre-frail, and Non-frail Elderly Patients Following Abdominal Surgery. *World J Surg* 2019; **43**:415-424.
- Stuck AE, Siu AL, Wieland GD, et al. Comprehensive geriatric assessment: a meta-analysis of controlled trials. *Lancet* 1993; **342**:1032-1036.
- Subramaniam S, Aalberg JJ, Soriano RP, et al. New 5-Factor Modified Frailty Index Using American College of Surgeons NSQIP Data. *J Am Coll Surg* 2018; **226**:173-181.e8.
- Subramaniam S, Aalberg JJ, Soriano RP, et al. The 5-Factor Modified Frailty Index in the Geriatric Surgical Population. *Am Surg* 2021; **87**:1420-1425.
- Susano MJ, Grasfield RH, Friese M, et al. Brief preoperative screening for frailty and cognitive impairment predicts delirium after spine surgery. Anesthesiology. 2020;133:1184–1191.
- Taraldsen K, Sletvold O, Thingstad P, et al. Physical behavior and function early after hip fracture surgery in patients receiving comprehensive geriatric care or orthopedic care--a randomized controlled trial. *J Gerontol A Biol Sci Med Sci* 2014;**69**:338–45.
- Tipping CJ, Hodgson CL, Harrold M, et al. Frailty in Patients With Trauma Who Are Critically Ill: A Prospective Observational Study to Determine Feasibility, Concordance, and Construct and Predictive Validity of 2 Frailty Measures. *Phys Ther* 2019; **99**:1089-1097.
- Torres-Perez P, Álvarez-Satta M, Arrazola M, et al. Frailty is associated with mortality in brain tumor patients*. Am J Cancer Res*. 2021;**11**:3294–3303.
- Tracy BM, Adams MA, Schenker ML, et al. The 5 and 11 Factor Modified Frailty Indices are Equally Effective at Outcome Prediction Using TQIP. *J Surg Res* 2020; **255**:456-462.
- Tracy BM, Carlin MN, Tyson JW, et al. The 11-Item Modified Frailty Index as a Tool to Predict Unplanned Events in Traumatic Brain Injury. *Am Surg* 2020; **86**:1596-1601.
- Tse W, Lavingia KS, Amendola MF. Using the risk analysis index to assess frailty in a veteran cohort undergoing endovascular aortic aneurysm repair. *J Vasc Surg*. 2022;**75**(5):1591-1597.e1.
- Valdatta L, Perletti G, Maggiulli F, Tamborini F, Pellegatta I, Cherubino M. FRAIL scale as a predictor of complications and mortality in older patients undergoing reconstructive surgery for non-melanoma skin cancer. *Oncol Lett*. 2019;**17**:263–269.
- Vidán M, Serra JA, Moreno C, et al. Efficacy of a comprehensive geriatric intervention in older patients hospitalized for hip fracture: a randomized, controlled trial. *J Am Geriatr Soc* 2005;**53**:1476–82
- Voskamp MJH, Vermeer M, Molijn G-J, et al. The Usefulness of the Modified Frailty Index for Muscle-Invasive Bladder Cancer Patients Treated with Radical Cystectomy. *Curr Urol* 2020; **14**:32-37.
- Wahl TS, Graham LA, Hawn MT, et al. Association of the Modified Frailty Index With 30-Day Surgical Readmission. *JAMA Surg* 2017; **152**:749-757.
- Wang HT, Fafard J, Ahern S, et al. Frailty as a predictor of hospital length of stay after elective total joint replacements in elderly patients. *BMC Musculoskelet Disord* 2018; **19**:14.
- Yajima S, Nakanishi Y, Umino Y, Ookubo N, Tanabe K, Kataoka M, Masuda H. Value of Geriatric Assessment Using the G8 to Predict Postoperative Urinary Tract Infections in Patients Undergoing Radical Cystectomy. *Turk J Urol*. 2022;**48**(4):278-286.
- Yamada S, Shimada M, Morine Y, et al. Significance of Frailty in Prognosis After Hepatectomy for Elderly Patients with Hepatocellular Carcinoma. *Ann Surg Oncol* 2021; **28**:439-446.
- Yin Y, Jiang L, Xue L. Comparison of three frailty measures for 90-day outcomes of elderly patients undergoing elective abdominal surgery. *ANZ J Surg*. 2021;**91**:335–340.
- Zennami K, Sumitomo M, Hasegawa K, Kozako M, Takahara K, Nukaya T, Takenaka M, Fukaya K, Ichino M, Fukami N, Sasaki H, Kusaka M, Shiroki R. Risk factors for postoperative ileus after robot-assisted radical cystectomy with intracorporeal urinary diversion*. Int J Urol.*  2022;**29**(6):553-558.
